# The Impact of Gender, Socioeconomic Status and Home Language on Primary School Children’s Reading Comprehension in KwaZulu-Natal

**DOI:** 10.3390/ijerph13030322

**Published:** 2016-03-15

**Authors:** Gabriela Völkel, Joseph Seabi, Kate Cockcroft, Paul Goldschagg

**Affiliations:** Department of Psychology, University of the Witwatersrand, Private Bag X3, Johannesburg 2050, South Africa; gabrielav@telkomsa.net (G.V.); Kate.cockcroft@wits.ac.za (K.C.); Paul.goldschagg@wits.ac.za (P.G.)

**Keywords:** reading comprehension, gender, socioeconomic status, language, South Africa

## Abstract

The current study constituted part of a larger, longitudinal, South African-based study, namely, The Road and Aircraft Noise Exposure on Children’s Cognition and Health (RANCH—South Africa). In the context of a multicultural South Africa and varying demographic variables thereof, this study sought to investigate and describe the effects of gender, socioeconomic status and home language on primary school children’s reading comprehension in KwaZulu-Natal. In total, 834 learners across 5 public schools in the KwaZulu-Natal province participated in the study. A biographical questionnaire was used to obtain biographical data relevant to this study, and the Suffolk Reading Scale 2 (SRS2) was used to obtain reading comprehension scores. The findings revealed that there was no statistical difference between males and females on reading comprehension scores. In terms of socioeconomic status (SES), learners from a low socioeconomic background performed significantly better than those from a high socioeconomic background. English as a First Language (EL1) speakers had a higher mean reading comprehension score than speakers who spoke English as an Additional Language (EAL). Reading comprehension is indeed affected by a variety of variables, most notably that of language proficiency. The tool to measure reading comprehension needs to be standardized and administered in more than one language, which will ensure increased reliability and validity of reading comprehension scores.

## 1. Introduction

Seabi and his colleagues [1] considered the dearth of research on the detrimental effects of extreme noise levels on children’s cognitive processing and scholastic performance in South Africa. They conducted a longitudinal study of aircraft noise and its effects on primary school teaching and learning in Durban, KwaZulu-Natal, based on the premise of the European Road Traffic and Aircraft Noise Exposure and Children’s Cognition and Health (RANCH) project. The results thereof indicated that aircraft noise exposure does indeed negatively impact on children’s school activities, which affects how they cope, and confirmed that high levels of environmental noise are inversely related to reading abilities in primary school children. This being said, it is important to consider how, if at all, other variables affect a child’s scholastic performance.

Demographic information obtained in the aforementioned study [1] included gender, socioeconomic status and home language. Further exploration into these variables and whether they have an effect on reading comprehension would provide valuable insight into teacher’s instruction, potential interventions as well as general knowledge of the effects of noise on learning.

### 1.1. Reading Comprehension and Literacy in the South African Context

Reading comprehension is dependent on a range of basic language and cognitive skills [2]. Although South Africa lies third highest of the developing countries in Africa in terms of literacy rates, the country has been found to be lower than that of most other countries worldwide [3], and it is important to consider the factors as to why this is. According to the Department of Basic Education of South Africa, literacy is introduced in Grade R (children generally aged 5 years turning 6 years of age in that year), which can be considered the first year of formal schooling. South Africa is faced with a plethora of challenges, and education level and employment are among these. Literacy rates are lower in poor communities, and parents are often unable to read and write themselves. Literate parents have higher expectations for their child’s academic performance and, because of this, are more motivated to assist their child, see their child’s reports, and ensure that they read to their child [4]. Thus, a child who is part of a less privileged household has very little chance of being taught, assisted or even stimulated using books and shared reading by his or her parents, thereby placing them at risk for academic difficulties such as reading comprehension. Reading comprehension is a skill heavily dependent on language.

### 1.2. The Role of Language in Reading Comprehension

In terms of the effect of language on reading comprehension in a South African context, Seabi *et al.* (2012) [5] postulated that learners who learn in a second language would perform poorly in comparison to those learning in their first language, given that the test of reading comprehension (SRS2) was an English-based assessment. English as an Additional Language (EAL) speakers may be at a double jeopardy when exposed to both noise and having to read and comprehend in their second language [5]. The results of this study illustrated a significant difference in favor of EL1 learners, which suggests that reading comprehension is influenced by language, particularly if the assessment is not in one’s home language [5].

Further to the above South African findings, Webb [6] conducted research into English as a second language at a tertiary institution: the University of Pretoria. The study revealed that the use of a second language as a language of learning and teaching (LoLT) could be unfavorable to the academic growth of students as well as to the assessment of their progress, if the students in question do not have the proficiency in the LoLT. Following tertiary education, proficiency in the standard language, which is generally English in South Africa, is imperative and non-negotiable for professional occupations in today’s marketplace. Consequently, the need to take the role of language in academic development and assessment very seriously is evident [6]. Although this study is referring in particular to tertiary level academics, it can be postulated that the same applies for secondary and primary level schooling. Poor proficiency in the LoLT causes poor academic achievement and a poor foundation for cognitive development [6], be it at a primary, secondary or tertiary level.

Conversely to the aforementioned research findings, authors Hipfner-Boucher *et al.* (2015) [7] conducted research in Canada on narrative abilities that yielded results indicating that overall performance of EAL learners was indistinguishable from that of their EL1 counterparts. This study further divided the EAL learners into two groups based on parent reports of the language most often heard and spoken at home (EAL English language users and EAL minority language users). Although the current study did not control for the amount of English exposure to EAL learners, one needs to still consider that perhaps EL1 learners will not always necessarily outperform EAL learners on reading comprehension tasks. Genesee (2015) [8] further explored the myths about early childhood bilingualism and concluded that learning two languages simultaneously is as natural as learning one, and that children can acquire full competence in two languages that is comparable with that of monolingual children. Based on this, perhaps it can be postulated that the same can be said for reading comprehension performance between EL1 and EAL learners.

### 1.3. Social Disadvantage (Socio-Economic Status)

Social disadvantage can be described in numerous ways and is difficult to distinctly define. Frequent measures of socioeconomic status (SES) include parental education level (usually maternal), occupation (usually paternal), economic deprivation, for example, low family income and poverty, type of housing, and high usage of medical and social services [9]. In South Africa particularly, the following indicators are increasingly being used as measures of the level and depth of poverty: unemployment, food security, housing, basic services, education and health [8]. The international community classifies South Africa as a middle-income country, yet the scale and demographic profile of poverty still indicates that it is one of the countries with the greatest levels of inequalities among its citizens [8].

The poverty rate in South Africa currently stands at 71% for rural areas [10]. This rate is indicative of a lower SES, and is measured by the proportion of people in a particular group or area falling below the poverty line. This high rate is concentrated in poor Black African and Colored communities, mimicking that which occurred during the apartheid era. In terms of schooling, three in five children in poor households attend inconsistently. Given this inconsistent scholastic attendance, it can only be assumed that lower academic achievement rates exist among those socially disadvantaged, and this is indeed confirmed by Statistics South Africa [11], which established that while 70.7% of all “Whites” in South Africa have at least a Grade 12 qualification, only 22% of “Africans”, 23.4% of “Coloreds” and 49.8% of “Indians” have an equivalent qualification [12].

In addition to these statistics, Pretorius & Naudé [3] looked at children between the ages of five and a half and seven years, whose first language was Setswana (*i.e.*, one of the South African indigenous languages), and who resided and were schooled in an informal settlement in South Africa. The aim was to determine what factors might play a role in the poor literacy and numeracy performance of young South African learners in comparison to that of other countries. Every child in the sample was assessed individually in Setswana (test material was translated into Setswana). Results of this study indicated that these children are ill-prepared for formal education: They have inadequate literacy skill, poor sentence construction, poor syntax knowledge, and inadequate phonological (sound) development [3]. While the fact that language may certainly play a role in under-developed literacy skills, there is no doubt that the additional factor of being poor and disadvantaged is also linked to poor cognitive and reading comprehension competency.

Further to this, there seems to be an interaction between SES and language and their effect on reading comprehension [7,13]. Although the current study did not take this into account, it is important to keep in mind given the fact that South Africa is a developing and middle-income country with 11 official languages, and that the interaction between socioeconomic inequalities and multilingualism indeed affects a child’s academic performance, particularly in literacy, as identified above.

### 1.4. Gender

In terms of reading, this is typically considered an activity whereby mothers play a more active role in teaching children to read and engaging in shared reading [14]. Boys and girls have been shown to differ in their motivation to read, reading choice, frequency thereof, attitudes towards reading, competency and the value of reading [14].

Taking into consideration reading and reading comprehension performance, a study conducted in the United States indicated that boys and girls begin grade one with approximately equal reading scores; however, as they grow up, a significant gender gap develops [4]. By grade 5, girls score considerably higher on reading tasks than boys. In addition to gender differences, the authors also considered SES, and found that the gender gap in reading seems to be characteristic mainly of children from socially disadvantaged families.

A similar longitudinal study conducted by Burbridge, as cited in Entwisle *et al.* [4], found an association between gender gap in reading and family SES and reported that boys in low SES households are far more likely than girls to be held back in school. However, a study conducted by Bianchi indicated slightly different results: In poverty-stricken families, boys are more likely than girls to be above the grade average, but as the poverty level decreases to better-off families, boys and girls are similarly likely to be above the grade average.

Many international studies, which included 35 to 40 countries, examined reading comprehension with 10-year-old children and found gender differences favoring girls in every participating country [14]. Further to this, the research shows that these differences in reading competence continue into adolescence and that gender differences appear regardless of the type of reading instruction children have received, or of the writing system. Girls and boys tend to differ in their reading preferences, habits and reading interests. Girls tend to read more and have better reading ability [15].

All these findings are important to keep in mind when considering reading comprehension performance on a standardized measure, and more research into the reading habits and preferences needs to be conducted within a South African context, as there is a dearth of information thereof. Although this study did not aim to look into this, it is important to keep insights from previous research in mind when considering gender difference performances on reading comprehension tasks.

### 1.5. Research Questions

This study was undertaken to answer the following questions:

1. What is the effect of gender on reading comprehension?
2. What is the effect of socioeconomic status on reading comprehension?
3. What is the effect of language on reading comprehension?

## 2. Method

### 2.1. Context of the Study

This study constituted part of a larger, longitudinal, South African-based study, namely, The Road and Aircraft Noise Exposure on Children’s Cognition and Health (RANCH-SA) study. RANCH-SA is based on the original RANCH project that primarily investigated the effects of aircraft and road traffic noise on children’s cognitive performance. The RANCH-SA project attempted to determine the effects that aircraft noise has on South African primary school children’s reading comprehension, attention, working memory and episodic memory in KwaZulu-Natal [16].

The RANCH-SA study administered five instruments. These included the following: the Suffolk Reading Scale Level 2 (SRS2) to assess reading comprehension, the Toulouse Pieron test that assesses attention, the Children’s Memory Scale, the Search and Memory task, and the Figure Analogies subtest of the Quantitative Battery for Cognitive Abilities test to assess IQ levels. No hearing tests were performed to screen for hearing impairments; however, parents were asked if their child(ren) had any known hearing difficulty [15].

For the purpose of this study, archival records were utilized, whereby only the effects of demographic variables on reading comprehension were investigated.

### 2.2. Research Design

This quantitative study employed a cross-sectional, archival design (as part of a longitudinal study) whereby observations of the same variable at one specific point in time are made. Archival studies, such as this study, make use of previously collected data for new analysis and to answer current research questions [17].

### 2.3. Participants

Permission to conduct the study was obtained (MED/11/0061H) from the Wits Human Research Ethics Committee (Non Medical). The sample was drawn from the larger RANCH-SA study. In total, 834 (*n* = 834) learners across 5 public schools in the KwaZulu-Natal province participated in the study. Participants were primary school learners in the age range of 8–14 years old from different grades. The mean age was 11 years 1 month. The sample consisted of 322 (39%) males and 331 (40%) females (*n* = 331). The gender of 181 (21%) learners was unknown.

### 2.4. Instruments

While five instruments were administered as part of the RANCH-SA study, only two instruments were utilized for this study. A biographical questionnaire was used to obtain relevant biographical data relevant to this study, and the Suffolk Reading Scale 2 was used to obtain reading comprehension scores.

#### 2.4.1. Biographical Questionnaire

The biographical questionnaire aimed to gather information regarding the participants’ home language, age, gender, health, support at home and school work. Socioeconomic status was also established from the questionnaire and was determined by whether or not a participant (child) was entitled to receive free meals at school. A significant correlation has been shown between free school meal ratio and a range of census indicators representative of socioeconomic status [16]. Thus, receipt of a free meal was linked to whether a participant’s caregiver was receiving a government social grant. The questionnaire was administered in English and completed prior to the assessment.

#### 2.4.2. Suffolk Reading Scale 2

Reading comprehension was measured using the Suffolk Reading Scale 2 (SRS2). It consists of 86 multiple choice sentence completion questions, each with five possible answers. The SRS2 was standardized in the United Kingdom using a sample of primary school children that was representative and mostly randomly selected. It has three comparable levels covering an age range from 6 years to 14 years 11 months. Each level has two parallel forms for easy testing arrangements.

Research was conducted in order to determine whether the SRS2 is a reliable instrument in the South African context [18]. This research was conducted on primary school learners in KwaZulu-Natal, South Africa, and formed part of the RANCH-SA study by Seabi and his colleagues. Results indicated that the test is a reliable measure of reading comprehension in the South African context despite its having been developed and standardized in the United Kingdom. The SRS2 proved to have a suitable internal consistency Cronbach alpha coefficient of 0.93, which was consistent with findings of the SRS2 in the United Kingdom, which revealed correlation estimates of above 0.7 (*r* = 0.85) [18]. Raw of the SRS2 were used as it was not standardized for the South African population. Further details about applicability of the SRS2 to the South African context is provided in another study [1].

### 2.5. Data Analysis

The data analysis in this study was performed using the Statistical Package for Social Sciences (SPSS). Descriptive (mean scores and standard deviation) and inferential statistics were utilized to analyze the data set. Independent *t*-tests were conducted, as all assumptions of normality had been fulfilled.

## 3. Results

### 3.1. Descriptive Statistics

The total data set consisted of 834 participants. These participants were from both noise-exposed and quieter environments. Descriptive statistics for the Suffolk Reading Scale 2 (SRS2), which measured reading comprehension, are presented in Table 1. Out of the total data set, 142 participants (17%) did not complete the SRS2; thus, a total of 692 (83%) of the scores were analyzed.

Table 2 shows the mean scores of reading comprehension for the three variables of this study: gender, socioeconomic status (SES) and language.

### 3.2. The Effects of Gender on Reading Comprehension

The first research question sought to determine the differences in reading comprehension performance between males and females. Results indicated that the difference in reading comprehension scores were not statistically different. This suggests that the learners’ gender does not influence performance on reading comprehension tasks.

### 3.3. The Effects of Socio-Economic Status on Reading Comprehension

The second research question aimed to examine the differences in reading comprehension performance between learners from a low socioeconomic status and those from a high socioeconomic status. The results presented in Table 3 indicate that learners from a low socioeconomic background (*M* = 34.9, *SD* = 15.3) performed significantly better than those from a high socioeconomic background (*M* = 30.8, *SD* = 14.2), *t*(465) = 3.07, *p* < 0.001, *d* = 0.28. An effect size of *d* = 0.28 indicates a weak effect size. This illustrates that, although socioeconomic status plays a role in the performance of reading comprehension, it does not play as significant a role as that of language, which indicated a moderate effect size of *d* = 0.53. However, another study indicated that the effect of socioeconomic status was only significant in this particular year of testing (2009); thereafter, there was no difference in reading comprehension performance between those learners who had a low socioeconomic status and those who did not [19]. In 2010, no effect size (*d* = −0.0001) was determined, and in 2011 a minimal effect size (*d* = 0.12) for socioeconomic status on reading comprehension was determined [19].

### 3.4. The Effects of Language on Reading Comprehension

Lastly, this study aimed to investigate the differences in reading comprehension performance between English as a First Language (EL1) *versus* English as Additional Language (EAL) speakers. As previously mentioned, the SRS2 is an English assessment tool; thus, it was assumed that EL1 learners would perform better than EAL learners. This assumption was correct as EL1 speakers had a higher mean reading comprehension score (*M* = 36.2, *SD* = 14.9) than EAL speakers (M = 28.6, *SD* = 13.5), *t*(658) = 6.86, *p* < 0.001, *d* = 0.53. This suggests that learners perform better on reading comprehension tasks that are administered and completed in their native language. An effect size of *d* = 0.53 indicates a moderate effect size, which suggests that language undoubtedly influences reading comprehension performance. Of further note is that the performance of EL1 learners is above that of the sample mean score (*M* = 31.3). Thus, this difference in means highlights the observation that reading comprehension performance of EL1 speakers generally exceeds the performance of the sample size as a whole.

## 4. Discussion

### 4.1. The Effects of Socio-Economic Status on Reading Comprehension

The second research question considered whether socioeconomic status (SES) affected children’s reading comprehension. The result indicated that children from a lower SES performed better than those from a high SES. However, it is of vital importance to note that this was the case for only the first year of testing; thereafter, there was no difference in reading comprehension performance between those learners who had a low SES and those who did not [19]. It was suggested that this may have been due to the fact that the participants who constituted the lower SES bracket in 2009 came from areas where there were high levels of noise, thus demonstrating the students’ potentially better adaptability and concentration, and in turn leading to better performance results [19]. These noise levels were as a result of the school’s being located in close proximity to the Durban International Airport in 2009. Although one would expect the opposite effect of noise exposure, the results could indicate that the children were more habituated to the extraneous noise experienced and thus better able to concentrate during the administration of the SRS2 [19].

As previously mentioned, there is generally a negative correlation of cognitive and academic achievement with SES. However, the result of this study challenges this notion, and is inconsistent with previous findings of that of Korat [20], Gentaz *et al.* [21] and Pienaar *et al.* [22], who found that learners from a lower SES achieved lower reading and writing scores than those from a higher SES. In lieu of these findings, the result of this study needs to be carefully considered.

One needs to consider, however, that perhaps the effect of socioeconomic status on reading comprehension was not yet apparent in the current study, and that this may become evident at a later stage in schooling, similarly to that of a previous study by Akhtar and Niazi [23], which confirmed that lower class students perform poorly at a secondary (high school) level of schooling. Likewise, although the findings of Jednoróg *et al.* [24] indicated an association between reduced academic performance and SES on neural imaging, perhaps this also only takes effect later on in schooling years. Lastly, although Pienaar and colleagues [22] concluded there was a negative association between low SES and academic performance, this study only included first grade learners, which is limiting when considering that primary school children can vary in age from 6 (grade one) to 13 years (grade seven), and that reading (comprehension) proficiency increases as a child gets older.

The quoted statistic (that three in five children in poor households attend school inconsistently) needs to be considered since those who participated in this study may not be part of those three children, but rather the two who do attend consistently. This being said, although the learner is from a low SES, he/she may be someone who is dedicated to school and works towards positive academic achievement, and thus performed well on the reading comprehension task.

The discussion above may explain why children of a lower SES performed better than children from a higher SES. However, it is important to mention that, although socioeconomic status plays a role in the performance of reading comprehension, and that this variable requires further investigation, it does not play as significant a role as that of language, which is yet to be discussed.

### 4.2. The Effects of Language on Reading Comprehension

This study aimed to determine the effect of language on children’s reading comprehension, with specific reference to having English as a First Language (EL1) in comparison to English as an Additional Language (EAL). The Suffolk Reading Scale 2 was administered in English. Language plays an integral role in the assessment of cognitive functioning, particularly that of reading comprehension. Testing, of any form, in one’s first language is imperative for the results to be truly reflective of a child’s abilities. Tests are generally standardized, which means that it allows for the ranking of a child’s performance on the test against that of typically developing children in the same age group [25]. The result was in favor of EL1 learners, which confirms the paradigm that testing in one’s native language is positively correlated to better reading comprehension. Furthermore, this supports the notion that formal standardized norm-referenced language tests are biased against children from culturally and linguistically diverse backgrounds because these children are not included in the normative sample [25]. Scores obtained from testing a South African child with a non-South African test would not be a valid indicator of the child’s abilities. Testing comprehension in a second language, regardless of the child’s perceived fluency, would not indicate their true abilities, and there would be no equal ground for different learners in understanding and constructing meaning from a text [25]. This is rife in South Africa in general.

Thus, had children been tested in their native language, or had the entire sample been made up of EL1 speakers, perhaps the results would have been different. However, given the multicultural and multilinguistic nature of South Africa and its youth, it can be expected that testing in one’s native language will not always be achievable, and that the result of this particular aim is debatable.

Until an assessment tool has been developed, which caters for the language needs of those whose first language is not English, it will be difficult to ascertain genuine reading comprehension outcomes, as this task is heavily dependent on language. The slow implementation of policy to introduce native languages as the language of learning and teaching (LoLT) by the Department of Education continues to place EAL learners at risk; thus, language remains a contentious issue in South Africa and for the primary, secondary and tertiary education, the latter of which is described by Webb [6], of its youth.

### 4.3. Strengths and Limitations

The findings of this study provide awareness into the possible effects of personal and sociocultural factors on reading comprehension. The reasonably large sample size allowed for generalizability and observed significant differences; however, the provision of Cohen’s *d* effect size balances this effect out. Investigation into the effect of language on reading comprehension revealed and highlighted the need for educational policy implementation, as well as the need for standardized testing tools to meet South Africa’s multilinguistic needs.

The results of this study should be read in the context of the following limitations. This study was cross-sectional in nature, and such studies do not allow for differentiation between cause-and-effect and simple association [26]. As a result of this, it cannot be said, for example, that gender causes impaired reading comprehension.

Students were not matched on age and/or IQ, which may contribute to increased variability on results and limitations on reporting these. Reading comprehension scores at only one point in time were analyzed and used to draw conclusions. Perhaps comparing scores at more than one point in time would provide more valuable information, *i.e.*, considering longitudinal data and discussing the changes over time. Lastly, although the language variable was differentiated as either EL1 or EAL, a variety of languages surely make up EAL, and thus generalizability of the study is limited.

### 4.4. Implications for Future Research

The findings of this study lend to suggestions for future studies in this field. The tool to measure reading comprehension, be it the SRS2 or an alternate tool, needs to be standardized and administered in more than one language, which will ensure increased reliability and validity of reading comprehension scores. Further to this, perhaps the language variable needs to be dissected into every single language spoken by the learners, and comparisons made on reading comprehension performance between these languages. Considering the conundrum of the combined influence of gender and SES on reading comprehension, further research into the interplay of these variables can be considered.

In order to determine the effects of gender, socioeconomic status and language on reading comprehension over time, further investigation into each of these variables at all three points in time of measurement should be done.

## 5. Conclusions

This study aimed to examine the impact of gender, socioeconomic status and language on the cognitive skill of reading comprehension in a sample of primary school learners in KwaZulu-Natal. The results of this research indicate that reading comprehension is indeed affected by a variety of variables. Although each variable was studied in isolation, there is no doubt that the interplay between them may also influence reading comprehension performance. One cannot consider that they are independent of each other.

It is clear and evident that language plays the most significant role in reading comprehension performance. Given the diversity of cultures and languages in South Africa, serious consideration needs to be given to the standardization and normalization of assessment tools. In terms of gender influencing reading comprehension performance, it is clear that females generally perform better, although not significantly so. Lastly, socioeconomic status attested to be a factor in performance, although the results thereof were unexpected, and further investigation into this may provide additional insight as to why this was so.

## Figures and Tables

**Table 1:** Descriptive statistics for the Suffolk Reading Scale 2 (SRS2). Reading comprehension descriptive statistics.

	*N*	Mean	*SD*
Reading comprehension	692	31.3	15.7

**Table 2:** Descriptive statistics for the three variables of this study. Reading comprehension scores of gender, SES and language groups.

Variable	*N*	Mean	*SD*
Male	332	30.6	15.3
Female	338	32.2	15.3
Low SES	257	34.9	15.3
High SES	338	30.8	14.2
EFL	410	36.2	14.9
EAL	374	28.6	13.5

**Table 3:** Results of the Independent *t*-test analyses. The effect of noise on gender, SES and language.

Variable	*DF*	*t*	*p*
Gender	565	−1.25	0.21
SES	465	3.07	0.00 *
Language	658	6.86	0.00 *

Notes: * Indicates significance at the 0.05 level (*p* < 0.05).

## Abbreviations

The following abbreviations are used in this manuscript:

RANCH	The Road and Aircraft Noise Exposure on Children’s Cognition and Health
SRS2	Suffolk Reading Scale 2
SES	Socio-Economic Status
EL1	English as First Language
EAL	English as Additional Language
